# Supplementary material for: End Sequence Analysis Toolkit (ESAT) expands the extractable information from single-cell RNA-seq data
Source: Genome Res. 2016 Oct;26(10):1397–410. doi: 10.1101/gr.207902.116 (PMC5052061; doi:10.1101/gr.207902.116)
Supplement: Supplemental Material [file supp_gr.207902.116_Supp_Materials.tar.gz › ESAT-esat_v0.1/doc/html/classbroad_1_1core_1_1math_1_1_loess.html]

ESAT: broad.core.math.Loess Class Reference


ESAT


- Main Page
- Related Pages
- Packages
- Classes
- Files

- Class List
- Class Index
- Class Hierarchy
- Class Members

All Classes Namespaces Files Functions Variables Enumerator Pages

- broad
- core
- math
- Loess

Classes |
Public Member Functions |
Static Public Member Functions |
List of all members

broad.core.math.Loess Class Reference

Collaboration diagram for broad.core.math.Loess:

[legend]

|  |  |
| --- | --- |
| Classes | |
| class | **MA** |
|  | |

|  |  |
| --- | --- |
| Public Member Functions | |
|  | Loess (String name) throws IOException |
|  | |
|  | Loess (HashMap hash1, HashMap hash2) |
|  | |
|  | Loess (HashMap hash1, HashMap hash2, double bandwidth, double smoothing) |
|  | |
|  | Loess (HashMap hashA, HashMap hashM, boolean flank) |
|  | |
| double | log2 (double val) |
|  | |
| Map | getMap () |
|  | |
| Map | getFinalMValues () |
|  | |
| Map | getOriginalMValues () |
|  | |
| Map | getAMap () |
|  | |

|  |  |
| --- | --- |
| Static Public Member Functions | |
| static Vector | readDstFile (File dstFile) throws IOException |
|  | |
| static void | main (String[] args) throws IOException |
|  | |

## Constructor & Destructor Documentation

|  |  |  |  |  |  |
| --- | --- | --- | --- | --- | --- |
| broad.core.math.Loess.Loess | ( | String | *name* | ) | throws IOException |

Here is the caller graph for this function:

|  |  |  |  |
| --- | --- | --- | --- |
| broad.core.math.Loess.Loess | ( | HashMap | *hash1*, |
|  |  | HashMap | *hash2* |
|  | ) |  |  |

|  |  |  |  |
| --- | --- | --- | --- |
| broad.core.math.Loess.Loess | ( | HashMap | *hash1*, |
|  |  | HashMap | *hash2*, |
|  |  | double | *bandwidth*, |
|  |  | double | *smoothing* |
|  | ) |  |  |

|  |  |  |  |
| --- | --- | --- | --- |
| broad.core.math.Loess.Loess | ( | HashMap | *hashA*, |
|  |  | HashMap | *hashM*, |
|  |  | boolean | *flank* |
|  | ) |  |  |

## Member Function Documentation

|  |  |  |  |  |
| --- | --- | --- | --- | --- |
| Map broad.core.math.Loess.getAMap | ( |  | ) |  |

|  |  |  |  |  |
| --- | --- | --- | --- | --- |
| Map broad.core.math.Loess.getFinalMValues | ( |  | ) |  |

|  |  |  |  |  |
| --- | --- | --- | --- | --- |
| Map broad.core.math.Loess.getMap | ( |  | ) |  |

|  |  |  |  |  |
| --- | --- | --- | --- | --- |
| Map broad.core.math.Loess.getOriginalMValues | ( |  | ) |  |

|  |  |  |  |  |  |
| --- | --- | --- | --- | --- | --- |
| double broad.core.math.Loess.log2 | ( | double | *val* | ) |  |

Here is the caller graph for this function:

|  |  |  |  |  |  |
| --- | --- | --- | --- | --- | --- |
| static void broad.core.math.Loess.main | ( | String[] | *args* | ) | throws IOException |

static

Here is the call graph for this function:

|  |  |  |  |  |  |
| --- | --- | --- | --- | --- | --- |
| static Vector broad.core.math.Loess.readDstFile | ( | File | *dstFile* | ) | throws IOException |

static

---

The documentation for this class was generated from the following file:

- java/broad/core/math/Loess.java


---

Generated on Fri Aug 15 2014 15:10:53 for ESAT by  

 1.8.7
